# Effects of Inhibin A on Apoptosis and Proliferation of Bovine Granulosa Cells

**DOI:** 10.3390/ani10020367

**Published:** 2020-02-24

**Authors:** Huitao Xu, Adnan Khan, Shanjiang Zhao, Huan Wang, Huiying Zou, Yunwei Pang, Huabin Zhu

**Affiliations:** 1 Embryo Biotechnology and Reproduction Laboratory, Institute of Animal Sciences, Chinese Academy of Agricultural Sciences, Beijing 100193, China; xuhuitao104@163.com (H.X.); zhaoshanjiang@caas.cn (S.Z.); 17710734315@163.com (H.W.); zouhuiying@caas.cn (H.Z.); pangyunwei@caas.cn (Y.P.); 2 Key Laboratory of Animal Genetics, Breeding, and Reproduction, MARA, National Engineering Laboratory for Animal Breeding, College of Animal Science and Technology, China Agricultural University, Beijing 100193, China; dr.adnan93@cau.edu.cn

**Keywords:** granulosa cells, inhibin A, apoptosis, proliferation

## Abstract

**Simple Summary:**

The ovarian granulosa cells play a crucial role in oocyte nourishing, secreting hormones that create functional bidirectional crosstalk with the oocyte. During follicle development, granulosa cells replicate, secrete hormones, and provide a critical microenvironment for follicular growth. Proliferation and differentiation of granulosa cells are essential for normal follicular growth, development of the oocyte, ovulation, and luteinization. Inhibins negatively regulate the production and secretion of follicle-stimulating hormone from the anterior pituitary, controlling ovarian follicle development. This study was aimed to explore the cellular and molecular adaptation of bovine granulosa cells under different concentrations of inhibin A. For instance, several physiological traits (cell viability, apoptosis, mitochondrial membrane potential, and cell proliferation) were assessed under different concentrations (0, 20, 50, and 100 ng/mL) of inhibin A. Results depicted that high doses of inhibin A boosted cell viability, mitochondrial membrane integrity, and cell proliferation, while inhibiting apoptotic rate in bovine granulosa cells.

**Abstract:**

Inhibin A is well known for its inhibitory properties against follicle-stimulating hormone (FSH), released through a pituitary–gonadal negative feedback loop to regulate follicular development. Ovarian folliculogenesis, hormonal biosynthesis, and gametogenesis are dependent on inhibins, playing vital roles in promoting or inhibiting cell proliferation. The present study explored the physiological and molecular response of bovine granulosa cells (GCs) to different concentrations of inhibin A in vitro. We treated the primary GCs isolated from ovarian follicles (3–6 mm) with different levels of inhibin A (20, 50, and 100 ng/mL) along with the control (0 ng/mL) for 24 h. To evaluate the impact of inhibin A on GCs, several in vitro cellular parameters, including cell apoptosis, viability, cell cycle, and mitochondrial membrane potential (MMP) were detected. Besides, the transcriptional regulation of pro-apoptotic (BAX, Caspase-3) and cell proliferation (PCNA, CyclinB1) genes were also quantified. The results indicated a significant (*p* < 0.05) increase in the cell viability in a dose-dependent manner of inhibin A. Likewise, MMP was significantly (*p* < 0.05) enhanced when GCs were treated with high doses (50, 100 ng/mL) of inhibin A. Furthermore, inhibin A dose (100 ng/mL) markedly improved the progression of the G1 phase of the cell cycle and increased the cell number in the S phase, which was supported by the up-regulation of the proliferating cell nuclear antigen PCNA (20, 50, and 100ng/mL) and CyclinB (100 ng/mL) genes. In addition, higher doses of inhibin A (50 and 100 ng/mL) significantly (*p* < 0.05) decreased the apoptotic rate in GCs, which was manifested by down regulating BAX and Caspase-3 genes. Conclusively, our study presented a worthy strategy for the first time to characterize the cellular adaptation of bovine GCs under different concentrations of inhibin A. Our results conclude that inhibin A is a broad regulatory marker in GCs by regulating apoptosis and cellular progression.

## 1. Introduction

Ovarian granulosa cells (GCs) under the action of FSH [1] produce peptide hormones of the transforming growth factor beta (TGF-β) superfamily. These hormones, including the inhibins, i.e., inhibin α subunit (INHA), inhibin β subunits (INHβA and INHβB), and activins [2,3], have been studied in mammals, and their functions and modes of action show consistency among species [4]. Inhibins are also produced by ovarian theca and luteal cells, and their synthesis depends on the developmental stage of the follicle [5]. Of the various TGF-β superfamily peptides listed above, the two of them that have shown a particular ability as a diagnostic tool for assessing reproductive potentials are INHA and B [6].

Inhibin A is a gonadal hormone that acts in concert with estradiol to exert specific negative feedback to control FSH secretion by the pituitary gland [7]. Furthermore, inhibin encourages or suppresses cell proliferation via paracrine or autocrine manners, respectively [8]. Additionally, inhibins act in an antagonist fashion; in the presence of betaglycan, inhibin forms a high-affinity complex with the activin type II receptor, inhibiting cellular growth by suppressing the function of activin [9,10]. Moreover, the effect of inhibin on cell proliferation is not consistent among different cell types. For example, downregulation of inhibin A in cultured GCs promoted cell proliferation indexes and subsequently increased the progression of the cells from the G1 phase to the S phase of the cell cycle [11]. In mice, the deficiency of ovarian inhibin results in GC tumors [8]. Likewise, inhibin, activin, and estrogens probably play some role in the impairment of ovarian cell proliferation and apoptosis [12,13,14]. The regulation pattern of inhibin A in swine GCs and oocytes is well documented to be increased with follicle development. In addition, INHBB under the influence of miRNA-34a induced apoptosis in GCs [15,16].

Moreover, while much is now known about the effects of inhibin regulation on normal GCs in other species [15,16], to the best of our understanding, no attempt has been made so far to compare different concentrations of inhibin A with the physiology and function of bovine GCs. We hypothesize that, relative to control, GCs exposed to different concentrations of inhibin A will experience alterations both in physiological traits and expression of the key genes required for normal cellular function. Therefore, the current study was aimed to explore the cellular and molecular adaptation of bovine GCs under different concentrations of inhibin A.

## 2. Materials and Methods

### 2.1. GCs Isolation and Culture

Bovine ovaries were collected from a local abattoir and transported to the laboratory in thermally insulated bottles, containing sterile physiological saline with 100 U/mL penicillin and 0.1 mg/mL streptomycin, at 28–30 °C, within 2 h of harvesting. After washing with a warm 0.9% NaCl solution three times and rinsing in 70% warm ethanol for 30 s, ovaries were washed thrice with warm Dulbecco’s Phosphate-Buffered Saline (DPBS). For isolation of GCs, healthy follicles (3–6 mm) were aspirated using an 18-gauge sterile needle (B-Braun, Germany) and transferred into 15 mL conical centrifuge tubes (Corning, NY, USA). The follicular fluid containing cumulus–oocyte complexes (COCs) and GCs was filtered using a filter with a diameter of 40 μm, leaving COCs on the filter. The filtrate with GCs was then transferred into 15 mL conical centrifuge tubes, centrifuged at 1000×g for 5 min. To assess the viability, after washing twice with DMEM/F12 1:1 medium (Gibco, CA, USA), GCs were stained with a 0.5% trypan blue dye exclusion assay. To get the final concentration of 5 × 105 cells/mL, GCs were re-cultured in DMEM/F12 1:1 medium supplemented with 10% fetal bovine serum (Gibco, CA, USA), and cultured in 6-well plates at 37 °C and 5% CO2 under humidified air. Culture medium was replaced after 24 h. Before inhibin A treatment, the culture medium was replaced with a serum-free medium containing 20 ng/mL androstenedione (Aladdin, Shanghai, China).

### 2.2. Identification of GCs by Immunofluorescence

GCs were seeded in a 12-well plate for 24 h, mounted on microscope slides. After 24 h, the media was removed and washed with PBS two times, each time for 5 min. Adherent GCs were fixed for 20 min with ice-cold absolute methanol, then permeabilized for 5 min at room temperature with 200 μL 0.1% Triton X-100 (Sigma-Aldrich, St. Locus, USA) in PBS. GCs were washed 3 times with PBS and blocked for 1 h with 5% goat serum, followed by incubation with the primary rabbit anti-FSHR antibody (Santa Cruz, CA, USA) at a 1:500 dilution overnight at 4 °C. The following day, GCs were washed for 10 min in PBS and incubated in fluorescein isothiocyanate conjugated with goat anti-rabbit IgG secondary antibody (Boster, USA) diluted at 1:1000 for 2 h in the dark. Cells incubated with BSA were taken as a negative control. Furthermore, after washing two times with PBS, GCs were exposed to the nuclear probe propidium iodide (PI) (Sigma-Aldrich, St. Locus, USA) for labelling nuclear DNA. GCs were further washed with PBS 3 times and was examined under a fluorescence microscope (Olympus, Tokyo, Japan) for obtaining fluorescence images. Furthermore, cell Sens Standard software (Olympus, Tokyo, Japan) was used for merging the red and green fluorescence images.

### 2.3. Estimation of Cell Viability

To investigate the impact of inhibin A on GCs viability, an MTT cell proliferation and cytotoxicity assay kit was used (njjcbio, Nanjing, China) according to the manufacturer’s protocol. Briefly, a total of 1 × 10^4^ detached GCs were inoculated into 96-wells plates and suspended in 0.1 mL medium per well supplemented with different concentrations of inhibin A (0, 20, 50, 100 ng/mL) for 24 h. In addition, a 50 μL 1xMTT solution was then added to each well and incubated for 4 h. After the incubation time, the solution was replaced with 150 μL DMSO per well. An ELX microplate reader (BioTek, Winooski, USA) at 570 nm was used for the measurement of absorbance. Cell viability was expressed as a percentage of the control group (0 ng/mL).

### 2.4. Assessment of Mitochondrial Membrane Potential

Briefly, to investigate MMP of GCs in all treated groups, the MMP assay kit with JC-1 (Beyotime, China) was used. GCs were harvested by enzymatic digestion using trypsin and washed with preheated PBS three times. After collection, an MMP assay kit with JC-1 (Beyotime, China) was used for staining GCs. The stained cells were then counted by using a fluorescence activating cell sorter (FACS) Calibur flow cytometer (BD Biosciences, CA, USA). The data were analyzed by Flowjo software (version Win64-10.4.0).

### 2.5. RNA Extraction and Quantitative RT-PCR (qRT-PCR)

**Figure 1:**
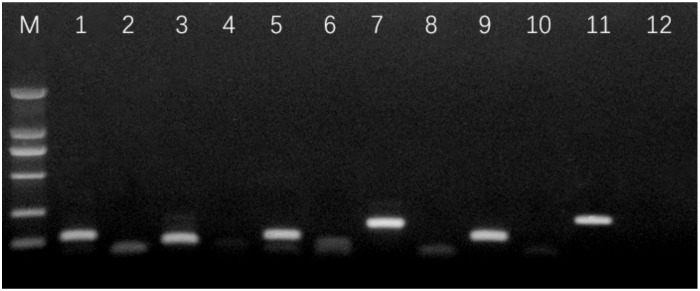
Amplification of six genes by gel electrophoresis. M: DL2000; 1: GAPDH; 2: GAPDH negative control; 3: Caspase-3; 4: Caspase-3 negative control;5: BAX; 6: BAX negative control; 7: PCNA; 8: PCNA negative control; 9: CyclinB1; 10: CyclinB1 negative control; 11: INHβA; 12: INHβA negative control.

Total RNA was extracted using an RNA kit (Hayue Yang Institute of Biotechnology, China) from three biological replicates of the control and inhibin A-treated GCs groups as described above, and was reverse transcribed to first-strand cDNA using a two-step RT-PCR kit (Takara, Dalian, China) according to the manufacturer’s protocols. The qRT-PCR was carried out under the 7500 fast real-time system (Agilent, CA, USA) using an RT-PCR kit (Takara, Dalian, China) as follows: preheating at 95 °C for 3 s; 40 cycles at 95°C for 5 s, and 60 °C for 34 s. At the end of each RT-PCR, the amplified product was subjected to a dissociation gradient to confirm the amplification of a single product by denaturation at the anticipated temperature. The expression levels were checked for six genes (BAX, Caspase-3, PCNA, CyclinB1, INHβA, and GADPH). Primer3 and web version 4.0.0 (http://bioinfo.ut.ee/primer3/) and primer blast (http://www.ncbi.nlm.nih.gov/tools/primer-blast/) were used designing gene-specific primers, and are shown in Table 1. The quality of the gene primers was checked by gel electrophoresis (Figure 1). RT-qPCR was performed using a Light Cycler 480 instrument (Roche, Germany). The data was acquired using the second derivative maximum method as computed by the Light Cycler Software 3.5 (Roche Diagnostics) and subjected for subsequent analysis. The 2−ΔΔCT method was used to calculate gene expression levels, using GAPDH as a reference gene.

### 2.6. Cell Cycle Analysis

The GCs’ cycle was detected 24 h after incubation with 100 ng/mL inhibin A using a cell cycle and apoptosis analysis kit (Beyotime, China). After incubation, GCs were harvested and washed three times with PBS. A minimum of 1 × 10^6^ cells was fixed in pre-chilled 70% ethanol at 4 °C overnight. Ethanol was then removed using centrifugation, and the GCs pellets were washed twice with 500 µL of 1 x PBS. Cellular DNA was stained with 50 µg/mL of propidium iodide (PI) and 50 µg/mL of RNase, being incubated at 37 °C for 30 min in the dark. Flow cytometry was performed by FACS Calibur (BD Biosciences, CA, USA), and data were analyzed by FlowjoV10 software.

### 2.7. Apoptotic Assay

The level of apoptosis in GCs collected from the indicated treatment groups (50 and 100 ng/mL) was detected using the Annexin V-FITC kit (Beyotime Biotechnology, China). GCs were harvested by enzymatic digestion using 0.25% trypsin after varying doses of inhibin A, and washed with preheated PBS. After collection, cells were stained with Annexin V-FITC following the manufacturer’s instructions. The stained cells were then counted by using a fluorescence activating cell sorter (FACS) Calibur flow cytometer (BD Biosciences, CA, USA). The data were analyzed by FlowjoV10 software.

### 2.8. Statistical Analysis

Data are expressed as mean values ± SEM. Statistical analysis was carried out using SPSS 16.0 and GraphPad Prism5 software (GraphPad Software Inc, San Diego, CA, USA). The differences between the control and treated groups were analyzed using a one-way ANOVA followed by multiple comparisons post-hoc tests. The treatment means were separated by Tukey’s test. Differences were considered to be statistically significant at *p* < 0.05.

## 3. Results

### 3.1. Effect of Inhibin A on GCs Identification and Viability

**Figure 2:**
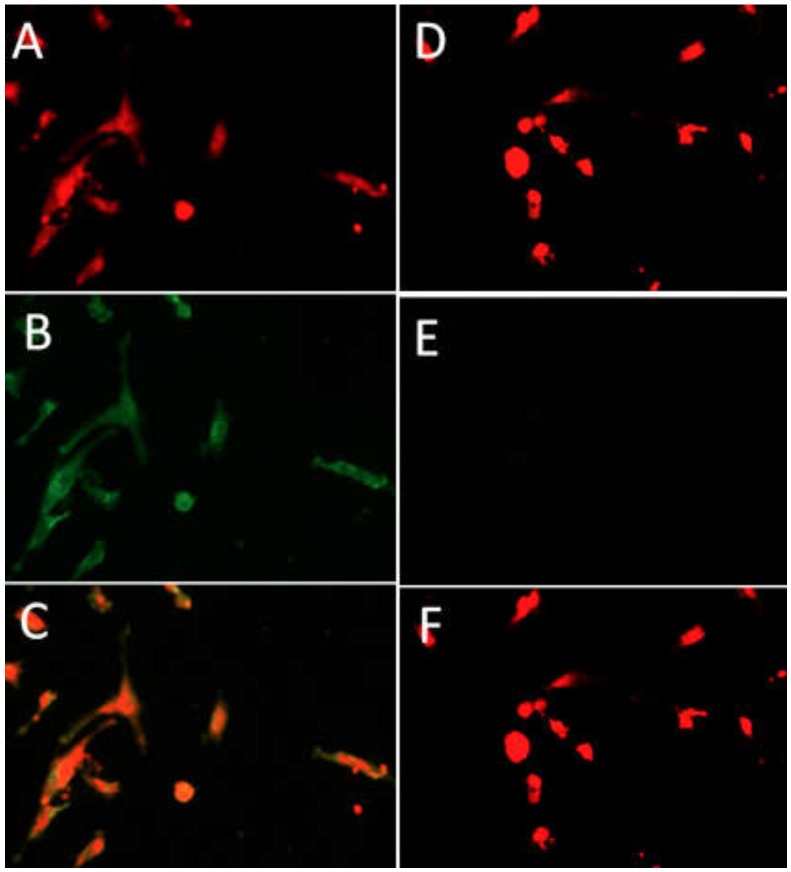
Granulosa cells (GCs) identification by immunofluorescence. PI-positive stained nuclei (**A**,**D**). FSHR-positive stained cells (**B**). (**E**) Negative control. (**C**) Image merging of (**A**) and (**B**); (**F**) Image merging of (**D**) and (**E**). A 50X magnification was used.

**Figure 3:**
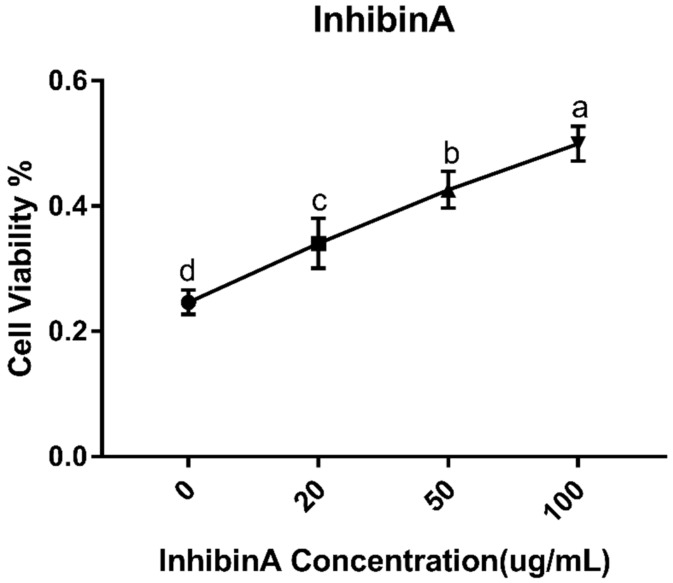
MTT assay of GCs cultured under different doses of inhibin A (20, 50, and 100 µg/mL) and the corresponding control (0 µg/mL). The measured cell counts for percent viability are indicated on the Y axis, and the doses of inhibin A are indicated on the X axis. Values are expressed as mean ± SEM of n = 3. The bars labeled with completely different letters indicate a significant difference, *p* < 0.05.

FSHR immunocytochemical staining was performed for the identification of GCs. The PI stain is localized in the nucleus, which is stained red, while the FSHR-positive staining resides in the cell membrane, and is stained green. Our result showed that under a microscope, more than 90% of the cells were FSHR positive, depicting that the purity of the GCs was above 90%, which were used for further experiments (Figure 2). The GC viability was estimated by MTT assay. It was found that the viability of the GCs was significantly higher (*p* < 0.05) in the inhibin A-treated groups than in the control (Figure 3). During cell culture, GCs were treated with a range of inhibin A doses (20, 50, and 100 µg/mL). Following treatment, the cell viability rate was significantly (*p* < 0.05) increased in a dose-dependent fashion.

### 4.2. Effect of Inhibin A on Mitochondrial Membrane Potential

**Figure 4:**
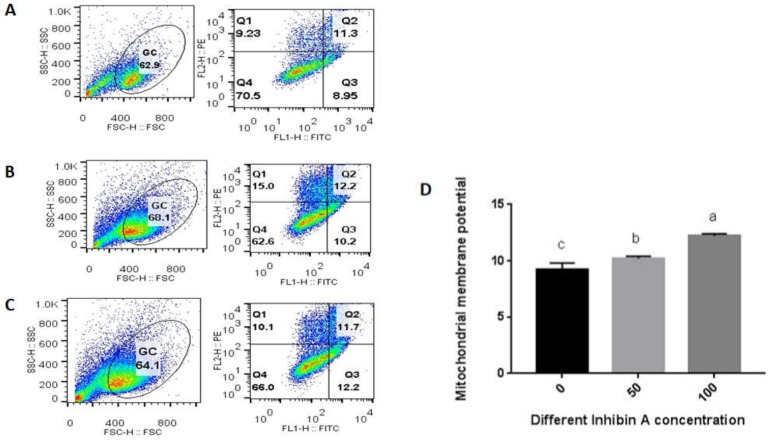
Flow cytometric analysis of GCs cultured under the treatments of different inhibin A doses (0, 50, and 100 µg/mL) (**A**–**C**), respectively. The analyzed cell counts for MMP are indicated on the Y axis and the doses of inhibin A are indicated on the X axis (**D**). Values are expressed as mean ± SEM of n = 3. The bars labeled with completely different letters indicate a significant difference, *p* < 0.05.

The MMP for GCs was measured by flow cytometry (FCM) to validate whether the apoptosis of the GCs was regulated by mitochondrial pathway. It was found that the MMP of the GCs was significantly higher (*p* < 0.05) in the inhibin A-treated groups (50 and 100 µg/mL) than the control group (0 µg/mL). Following the treatment, the MMP was significantly (*p* < 0.05) increased in dose-dependent fashion (Figure 4).

### 4.3. Effect of Inhibin A on Cell Cycle Progression

**Figure 5:**
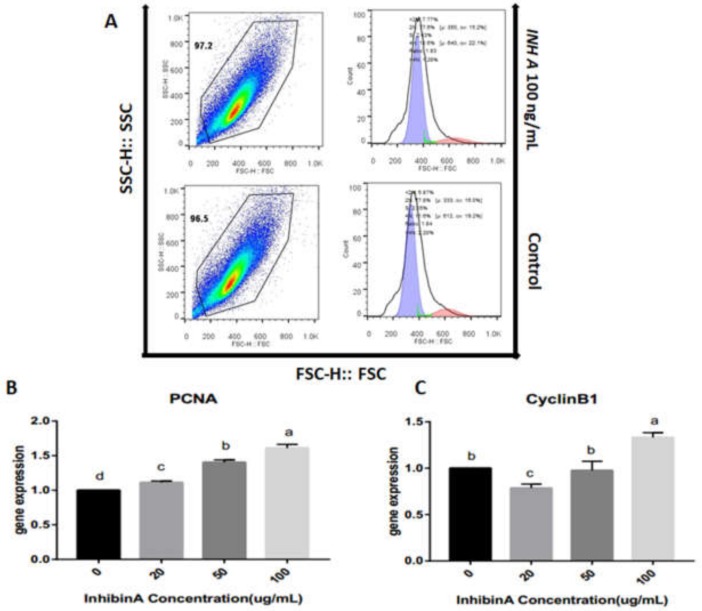
Inhibin A alters the stages of GCs cycle: Flow cytometric analysis of GCs cultured under the treatment of inhibin A (100 ng/mL) and the corresponding control group. The Y axis shows the analyzed cell counts while the X axis indicates the DNA content of cells stained by PI staining (**A**). mRNA expression of PCNA (**B**) and CyclineB1 (**C**) in GCs cultured under different concentrations of inhibin A (20, 50, and 100 ng/mL) and the corresponding control (0 ng/mL). GADPH was used as a reference gene. The results are expressed as the mean ± SEM, n = 3. The bars labeled with completely different letters indicate a significant difference, *p* < 0.05.

The effect of inhibin A in the GCs’ cycle was examined by flow cytometry (FACS Calibur, BD Biosciences, CA, USA). Our findings revealed that a higher dose of inhibin A (100 ng/mL) significantly (*p* < 0.05) increased the percentage of GCs in the S (DNA synthesis) phase at 24 h than in the control group (Figure 5A, Table 2). Likewise, the regulation of cell proliferation genes i.e., PCNA and CyclinB1, were also examined in GCs cultured under different doses of inhibin A (20, 50, and 100 ng/mL). It was observed that compared with the control group, the expression of PCNA increased significantly (*p* < 0.05) in a dose-dependent pattern (Figure 5B) while Cyclin B1 showed a significant (*p* < 0.05) increase at 100 ng/mL (Figure 5C).

### 4.4. Effect of Inhibin A on GCs Apoptosis

**Figure 6:**
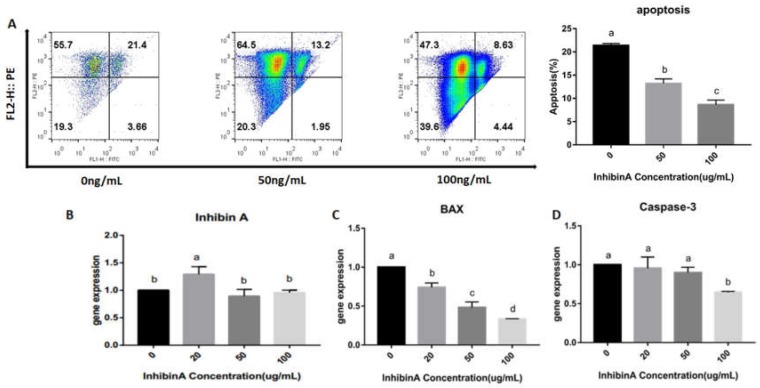
Flow cytometric analysis of GCs cultured under the treatments of different inhibin A doses (50 and 100 µg/mL). The Y axis shows the analyzed cell counts for apoptosis while the X axis indicates the doses of inhibin A DNA content of cells stained by PI staining. The analyzed cell counts for apoptosis are indicated on the Y axis, and the doses of inhibin A are indicated on the X axis (**A**). Data shown as means ± SEM, n = 3, *p* < 0.05. mRNA expression of INHβA (**B**) and pro-apoptotic genes BAX (**C**) and Caspase-3 (D) in GCs cultured at different concentrations of inhibin A (20, 50, and 100 ng/mL) and the corresponding control (0 ng/mL). GADPH was used as a reference gene. The results are expressed as the mean ± SEM, n = 3. The bars labeled with completely different letters indicate a significant difference, *p* < 0.05.

The apoptotic rate of the GCs was estimated by flow cytometry (FCM). It was found that the apoptotic rate of the GCs was significantly lower (*p* < 0.05) in the inhibin A-treated groups (50 and 100) than the control group (Figure 6A). Following inhibin A treatment, the cell apoptotic rate decreased significantly (*p* < 0.05) in a dose-dependent fashion. Furthermore, to investigate the impact of inhibin A on the induction of apoptosis in GCs, cells were examined for the regulation of INHβA and pro apoptotic genes; BAX and CASPASE-3. Our findings suggested that inhibin A induces the upregulation of INHβA at 20 ng/mL while no significant (*p* *<* 0.05) change was found at 50 and 100 ng/mL (Figure 6B). Moreover, downregulation of the BAX and Caspase-3 genes in GCs were noted at higher doses. However, Caspase-3 expression did not show a significant difference at 20 and 50 ng/mL when compared with the control (Figure 6C,D).

## 4. Discussion

**Figure 7:**
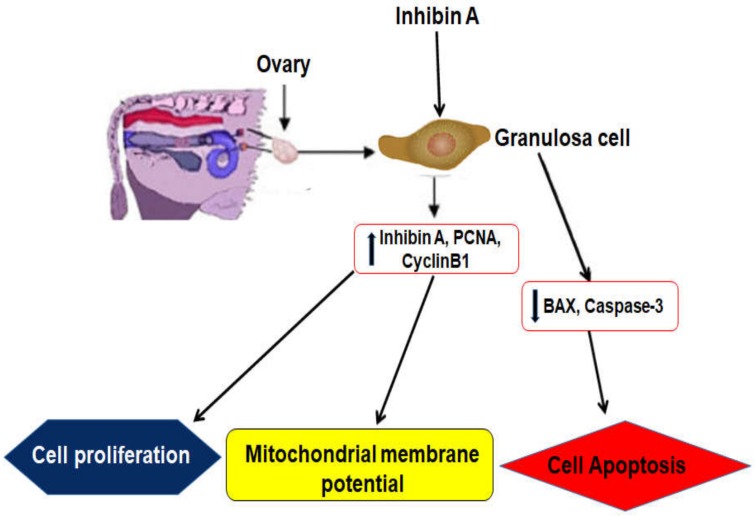
Mechanisms of regulating inhibin A response related to GC function within a bovine ovary. The upregulated genes inhibin A, PCNA, and CyclineB1 were involved in the regulating mechanism of GCs via boosting cell proliferation and mitochondrial membrane potential. Under inhibin A treatment, the downregulated genes BAX and Caspase-3 were involved in inhibiting GC apoptosis, which might enhance the possibility of GCs viability and follicle function.

Ovarian GCs play a crucial role in oocyte nourishing, secreting hormones that create functional bidirectional crosstalk with the oocyte [17]. Inhibin plays a significant role in the regulation of the whole reproductive axis and incorporates all reproductive events [18]. Ovarian follicles produce inhibin A and has a major inhibitory role against FSH secretion [9,19]. Furthermore, mutation of the INHβA gene also increased the risk of follicular cyst formation [20,21]. Therefore, inhibin A may promote cell proliferation under estradiol secretion. A brief overview of the current study and mechanisms of regulating inhibin A response related to follicular function within the bovine ovary is shown in Figure 7.

The versatile effect of the inhibin A gene provoked us to check its role in the regulation of apoptosis, cell cycle, and mitochondrial membrane potential regulations in GCs. Recently, inhibin A has been implicated in the maintenance of cellular development and apoptosis in different cellular models [11,22]. The current study examined the hypothesis that relative to a control, GCs exposed to different concentrations of inhibin A might experience alterations both in physiological traits and expression of key genes required for normal cellular functions. For instance, GCs were treated with different levels of inhibin A (20, 50, and 100 ng/mL) in vitro. It was found that compared to the control group, inhibin A significantly decreased apoptosis in GCs and thus had an important role in the suppression of programmed cell death in these cells. To further verify the apoptotic factors, we quantified the mRNA of apoptotic inducer (Caspase-3 and BAX) GCs. A significant decrease in the expression of Caspase-3 and BAX transcriptional levels were found in the inhibin A-treated groups. This indicated that inhibin A suppresses mitochondrial-mediated apoptosis [22]. Previous studies reported casp3-dependent repression of apoptosis in ovarian granulosa cells when treated with an inhibin analogue [18]. Caspase-3 is an important pro apoptotic gene, responsible for the induction of apoptosis in all types of cells. Its activation leads to the initiation of a cascade of caspases, responsible for the execution of cells [23].

Furthermore, we found that inhibin A promoted GC viability in a dose-dependent manner. Our data revealed that the proliferation of GC was significantly higher in the S cell cycle phase when treated with 100 ng/mL inhibin A. PCNA, CyclinB1, and INHβA are considered as key proteins involved in the proliferation and apoptotic pathway in GCs [24,25,26]. Moreover, PCNA and CyclinB1 are mainly expressed during the S phase of the cell cycle, and were used as a biomarker in the regulation of proliferation. Likewise, other proteins that regulate the cell cycle include CDK2 and CDK inhibitor p21, interacting with PCNA, and thereby affecting the progression of the cell cycle [26]. We quantified the relative mRNA expression of PCNA and CyclinB1. The results showed significant upregulation of PCNA and CyclinB1 in a dose-dependent fashion.

The disruption of MMP is a leading cause of cellular apoptosis and cell damage [27]. Mitochondria releases cytochrome c into the cystol when the function of the mitochondria is altered. Furthermore, the released cytochrome c triggers the regulation of Caspase-3, which is a key apoptotic factor, resulting in chromatin condensation and DNA fragmentation [28]. Our data suggested that a 100 ng/mL dose of inhibin A remarkably boosted the MMP of GCs. Likewise, inhibin A (50 and 100 ng/mL) significantly downregulated the expression of BAX and Caspase-3. Our results are consistent with findings reported by previous studies [29,30]. Our research can further be extended to understand the effect of inhibin A-treated GCs on bovine oocyte modulation and embryo development.

## 5. Conclusions

In the current study, we demonstrated for the first time a worthy strategy to characterize the cellular and transcriptional adaptation of bovine GCs at different concentrations of inhibin A (20, 50, and 100 ng/mL) in vitro. Furthermore, our results unveiled that inhibin A was important in the regulation of cell apoptosis, viability, MMP, and cell cycle progression from the G1 phase to the S phase in GCs. Hence, inhibin A can effectively improve the proliferation of GCs in vitro. This was further confirmed by the associative apoptotic and cell cycle factors.

## Figures and Tables

**Table 1:** List of primers used for qRT-PCR.

Gene	Accession no.	Forward 5′→3′	Reverse 5′→3′
**PCNA**	NM_001291925.1	GCGTTCATAGTCGTGTTCCG	TTCAAGATGGAGCCCTGGAC
**BAX**	XM_003355974.2	GGCTGGACATTGGACTTCCTTC	TGGTCACTGTCTGCCATGTGG
**CASP-3**	XM_005671704.1	TACTTGGGAAGGTGTGAGAAAACTAA	AACCCGTCTCCCTTTATATTGCT
**CyclinB1**	NM_001170768.1	AAGACGGAGCGGATCCAAAC	CCAGTGACTTCACGACCCAT
**INHβA**	NM_214189.1	GCTACCACGCCAACTACTGT	ACATGGGTCTCAGCTTGGTG
**GAPDH**	NM_001034034.2	GGTGCTGAGTATGTGGTGGA	GGCATTGCTGACAATCTTGA

**Table 2:** Cell cycle analysis.

Treatment	G0/G1	S	G2/M
Control	77.8 ± 0.2	2.05 ± 0.05 ^a^	11.6 ± 0.4
Inhibin A (100 g/mL)	77.6 ± 0.76	2.43 ± 0.13 ^b^	10.6 ± 0.2

Effect of inhibin A on cell cycle of GCs. Cell cycle analysis after 100 ng/mL inhibin A treatment compared with the control group. The superscripts with completely different letters indicate a significant difference, *p* < 0.05.
